# Septic Shock Due to Brevundimonas diminuta Bacteremia in an Immunocompetent Patient: A Case Report With a Literature Review of Antimicrobial Resistance Patterns

**DOI:** 10.7759/cureus.94921

**Published:** 2025-10-19

**Authors:** Aleksandra Kaplina, Kanika Rathi, Chandra Hassan, Michael Samotowka, Riley G Jones

**Affiliations:** 1 Internal Medicine, Bogomolets National Medical University, Kyiv, UKR; 2 Global Health, MedGlobal, Inc., Chicago, USA; 3 Division of Hospital Medicine, University of Florida, Gainesville, USA; 4 Division of General, Minimally Invasive and Robotic Surgery, University of Illinois, Chicago, USA; 5 Department of Surgery, Louis Stokes Cleveland VA Medical Center, Cleveland, USA

**Keywords:** antimicrobial resistance pattern, brevundimonas spp, emerging infectious diseases, fluoroquinolone resistance, gram-negative bacteremia, immunocompetent patients, septic shock (ss)

## Abstract

*Brevundimonas* species are a group of aerobic, non-fermenting, Gram-negative bacilli that are primarily environmental organisms commonly isolated from soil, water, and hospital surfaces. While historically regarded as non-pathogenic, they are increasingly recognized as opportunistic pathogens in immunocompromised or critically ill individuals. While most *Brevundimonas* species are not known to cause human disease, *Brevundimonas diminuta* and *Brevundimonas vesicularis* are the two species most commonly associated with human infections. Due to its uncommon occurrence and variable antibiotic susceptibility, *B. diminuta* is a diagnostic and therapeutic challenge. To our knowledge, only 12 cases of *B. diminuta *infections have been reported in North America to date. Here, we present a rare case of septic shock due to *B. diminuta* bacteremia in an immunocompetent patient.

## Introduction

*Brevundimonas* is a genus of bacteria comprised of 25 species of bacteria, which are rarely associated with human infections. *Brevundimonas* are aerobic, non-fermenting, Gram-negative rods with catalase- and oxidase-positive activity. Morphologically, they have a small, polar flagella [1]. *Brevundimonas* were first reported as human pathogens in 1993, although it was still classified at the time as a strain of *Pseudomonas *[2]; however, the incidences may be somewhat underreported since identification with real-time PCR was not widely available until 2010. Although rare, *Brevundimonas* has recently been identified as an emerging pathogen, primarily *B. vesicularis* and, to a lesser extent, *B. diminuta*. To our knowledge, only 12 cases of *B. diminuta* infection have been reported in the United States [3-8], with only three cases of bacteremia in adults or children [3,6,7], all of whom were reportedly immunocompromised. Importantly, *Brevundimonas* have a wide range of antimicrobial resistance, which, coupled with limited reports and data available, makes management difficult when clinically significant infections are encountered. Here, we present the full body of known resistance patterns of this rare disease by presenting this case of *B. diminuta* bacteremia causing septic shock in an immunocompetent patient who was successfully treated with cefepime and made a full recovery.

## Case presentation

A 64-year-old male with a history of coronary artery disease, hypertension, major depressive disorder, and alcohol use disorder presented with severe fatigue and malaise. He reported hospitalization one month prior for an episode of severe alcohol withdrawal; he had no history of immunosuppression or immunocompromising condition. From the time of discharge, he reported ongoing alcohol abuse accompanied by progressive weakness to the point of being bed-bound for the week leading up to hospitalization; he denied a history of IV drug use or vascular access devices. He was found by a maintenance worker on a routine house call and taken to the emergency department. 

On arrival, he was hypothermic to 35.4°C (95.8°F) and hypotensive to 80/41 mmHg with a pulse of 74 beats per minute and a respiratory rate of 18 breaths per minute. He was ill-appearing and found with a stage II sacral decubitus ulcer wound, which did not show any signs of infection; the remainder of his exam was normal. He did not meet the American Society for Parenteral and Enteral Nutrition (ASPEN) criteria for malnutrition. CT imaging of the head, chest, and abdomen was negative for acute findings. He was negative for HIV and hepatitis B and C. Laboratory results relevant to the case are presented in Table 1.

**Table 1:** Pertinent laboratory results

Laboratory tests	Patient's values	Reference range
Blood		
Sodium	122 mmol/L	136-145 mmol/L
C-reactive protein (CRP)	193.8 mg/L	0.0-5.0 mg/L
Procalcitonin	1.58 ng/mL	<0.1 ng/mL
White blood cells (WBC)	11.7 × 10E3/uL	4.0-10.0 × 10E3/uL
Lactic acid	1.9 mmol/L	0.3-1.5 mmol/L
Creatinine	5.87 mg/dL	0.51-1.18 mg/dL
Urine		
Leukocyte esterase	Positive	Negative
White blood cells per high-power field	21	0-5

His hypotension was refractory to multiple fluid boluses; thus, vasopressors were started, two sets of blood cultures were obtained, and broad-spectrum antibiotic therapy was initiated with vancomycin and cefepime. The patient was diagnosed with septic shock and admitted to the intensive care unit (ICU) for further management. Urine cultures revealed polymicrobial bacterial overgrowth of greater than three microorganisms and were deemed contaminated due to possible faulty collection by a laboratory testing protocol, consistent with a benign physical exam of the abdomen. His blood cultures were ultimately positive for high-grade *Brevundimonas diminuta* in four of four blood culture samples. Cultures revealed sensitivity to cefepime; thus, antibiotics were narrowed (Table 2). Repeat blood cultures after 48 hours were negative, so a one-week course of cefepime was administered. Immunoglobulin and vitamin levels were obtained and were normal. The infectious disease service was consulted for further exploration of the source of infection; however, no source or point of entry could be identified. He remained in the ICU for three days and had a steady recovery with normalization of renal function and inflammatory markers. He was transferred to the medical floor on hospital day 4, where he continued cefepime for a total of seven days. He was discharged home, and at the one-month follow-up, he remained well, with no recurrence of illness or bacteremia. At that encounter, he provided written consent for the publication of his case.

**Table 2:** Susceptibility report

Antibiotic	MIC	Interpretation
Amikacin	≤2 µg/mL	Susceptible
Cefepime	8 µg/mL	Susceptible
Ceftazidime	16 µg/mL	Intermediate
Ciprofloxacin	≥4 µg/mL	Resistant
Gentamicin	≤1 µg/mL	Susceptible
Levofloxacin	4 µg/mL	Intermediate
Meropenem	0.5 µg/mL	Susceptible
PiperacillintTazobactam	≤4 µg/mL	Susceptible
Tobramycin	≤1 µg/mL	Susceptible
Trimethoprim/sulfamethoxazole	≥320 µg/mL	Resistant

## Discussion

The genus *Brevundimonas *was first proposed by Segers et al. in 1994 by identifying *Pseudomonas diminuta* and* Pseudomonas vesicularis* as a distinct genus based on the results of DNA-rRNA hybridization studies [2]. Subsequent quantitative real-time polymerase chain reaction (qPCR) and fluorescence in situ hybridization (FISH) assays were developed in 2010, making the identification of *B. diminuta* more widely and rapidly available. 

A number of severe infections in malnourished and immunocompromised individuals have been associated with* Brevundimonas,* such as Noma disease, alternatively known as cancrum oris, a rapidly progressive orofacial gangrene typically affecting severely malnourished children in low-resource settings. While our patient was physically debilitated, evaluation by a clinical nutritionist confirmed the absence of malnutrition, and serum analysis with normal levels of vitamins B1, B5, B6, B9, B12, D3, C, Zn, Se, and thyroid-stimulating hormone. In addition, he was not considered immunocompromised, as his immunology laboratory results were within normal limits, no history of using immunosuppressing agents, and his HIV status was negative. His low heart rate of 74 beats per minute was attributed to beta-blockade used for the patient’s coronary artery disease and hypertension management. 

**Figure 1:**
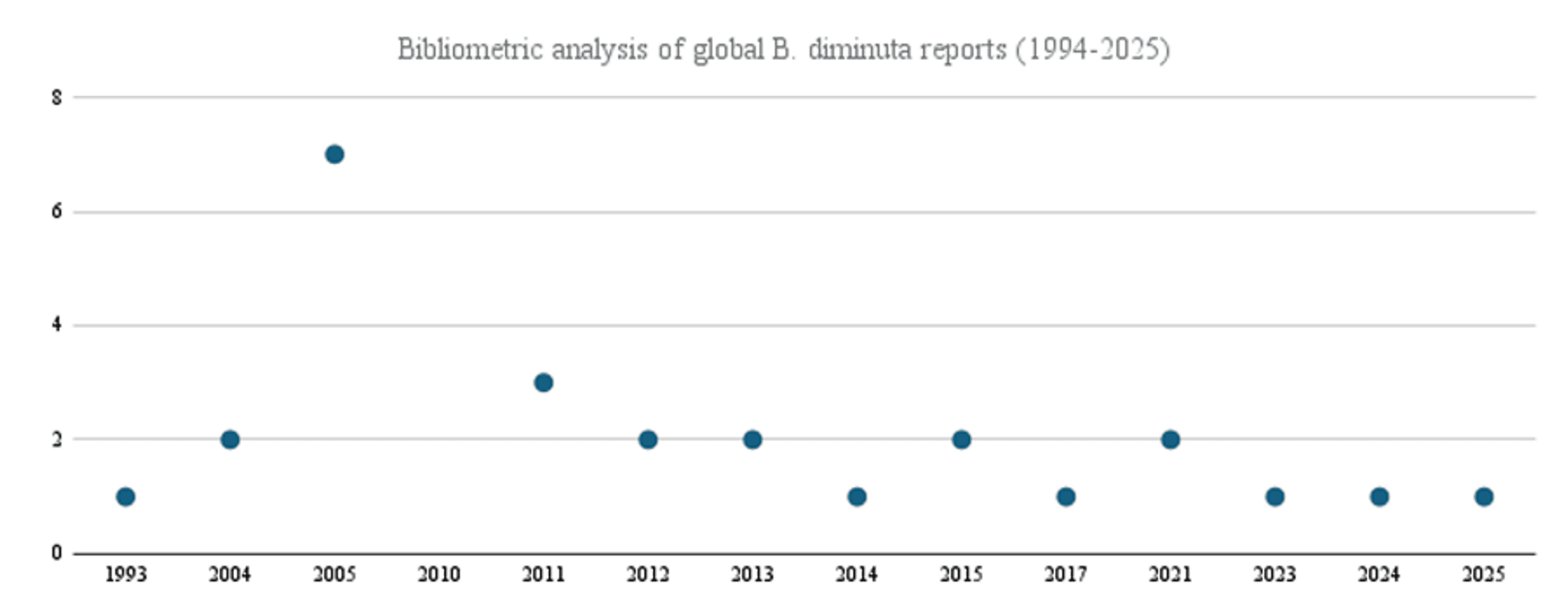
Bibliometric analysis of global Brevundimonas diminuta reports (1994-2025)

Infections caused by *B. diminuta* are rare and have been infrequently reported in the medical literature; its virulence is generally considered low [1]. As a result, there is limited understanding of the pathogen's full clinical significance, pathogenic mechanisms, and optimal treatment strategies, which poses a challenge to clinicians faced with a clinically significant infection. Using a PubMed, EMBASE, and CINAHL query with citation chain search of "brivundimonas", "brivundimon*", and "pseudomonas diminuta" were captured and filtered for human reports, wherein 26 cases of *B. diminuta* infections worldwide out of 27 with citations were able to be located. Sixteen cases were reported across 10 reports after 2010 (Figure 1), which might correlate with more easily accessible testing and increased recognition of the species. Most of the reports were complicated by significant comorbidities that rendered patients immunocompromised and susceptible to opportunistic infections; only five cases were observed in immunocompetent patients without significant comorbidities (Table 3). Our analysis revealed an important complication in the existing literature: a widely cited paper was found with significant discrepancies between the manuscript text and the supplemented chart [1]. Furthermore, a single report in grey medical literature was unable to be obtained for review despite exhausting attempts with the assistance of a research librarian.

**Table 3:** Brevundimonas diminuta infections reported worldwide (1993-2025) N/A - not applicable, UTI - urinary tract infection, DM - diabetes mellitus, CLL - chronic lymphocytic leukemia, COPD - chronic obstructive pulmonary disease, CHFpEF - chronic heart failure with preserved ejection fraction, NASH - non-alcoholic steatohepatitis, OSA - obstructive sleep apnea

Author (Ref.)	Year	Sex/age	Country	Comorbidity	Type of infection
Pasadakis et al. [9]	1993	N/A	Greece	End-stage renal failure	Peritonitis
Seve et al. [10]	2004	F/35	France	Leukemia	Bacteremia (hospital-acquired)
Chi et al. [11]	2004	M/62	Taiwan	Liver cirrhosis (hepatitis B associated), esophageal varices, encephalopathy, spontaneous bacterial peritonitis, duodenal ulcer	Bloodstream infection (community-acquired)
Han et al. [3]	2005	Multiple (7 cases)	USA	Cancer	Bacteremia, UTI, empyema (hospital-acquired)
Lee et al. [12]	2011	3 Cases	Taiwan	Cancer	Bacteremia
Almuzara et al. [13]	2012	F/56	Argentina	Lupus glomerulonephritis	Leg ulcer
Pandit et al. [4]	2012	F/66	USA	N/A	Keratitis (community-acquired)
Lu et al. [14]	2013	M/38	China	None	Pleuritis
Shobha et al. [15]	2013	Infant	India	None	UTI
Mahapatra et al. [16]	2014	M/35	India	None	Post-traumatic abscess
Cao et al. [17]	2015	M/62	China	Myelodysplastic syndrome, DM type 2	Bacteremia
Ghosh et al. [18]	2015	N/A	India	Tuberculosis	N/A
Chandra et al. [19]	2017	M/18	India	Focal segmental glomerulosclerosis with nephrotic syndrome	Bacteremia
Lupande-Mwenebitu et al. [20]	2021	Newborn	Democratic Republic of Congo	Low-weight, preterm	Omphalitis
Burch et al. [5]	2021	M/67	USA	DM type 1, stage 0 CLL	Hepatic abscess
Hassan et al. [6]	2023	M/47	USA	COPD, bipolar disorder, stable seizure disorder	Sepsis, lung abscess
Ferreira Caceres et al. [7]	2024	M/69	USA	Adrenal insufficiency secondary to panhypopituitarism, CHFpEF, atrial fibrillation, NASH, OSA, pulmonary hypertension, morbid obesity	Sepsis secondary to cellulitis of the abdominal wall and right lower extremity
Thareja et al. [8]	2025	M/50	USA	N/A	Keratitis

Given the rarity of* B. diminuta *as a clinical pathogen, initial consideration was given to the possibility of specimen contamination; however, this was determined less likely when the second set was positive with concordant antibiotic resistance patterns. Alternatively, a laboratory pseudo-outbreak during the evaluation was considered. Pseudo-outbreaks are defined as an increase in identified organisms without evidence of true infection, usually reflecting laboratory supply contamination. They have been linked to factors such as contaminated culture media, syringes, blood culture vials, inadequate disinfection of analyzers, and improperly sterilized equipment. The literature review only shows two episodes of pseudo-outbreaks, one in the United States in 2017 and in South Korea in 2011 [1]. In our case, consultation with hospital infection control staff confirmed no pseudo-outbreak in our facility, further highlighting *B. diminuta* as the cause of septic shock in our patient.

Antimicrobial resistance is an emerging concern worldwide. In our analysis, *B. diminuta* showed varied and extensive resistance patterns. Concerningly, *B. diminuta* appeared widely resistant against antibiotics commonly used as empiric treatments for various infections, such as sepsis and abscesses, namely, fluoroquinolones, sulfonamides, colistin, and several beta-lactams. Antibiotic sensitivity is conserved with carbapenems, aminoglycosides, and ticarcillin/clavulanate. As such, the sensitivity analysis should be performed on a case-by-case basis to tailor antibiotic regimens when *B. diminuta* infections are encountered (Table 4).

**Table 4:** Brevundimonas diminuta antimicrobial resistance patterns S - sensitive, R - resistant, I - intermediate

	Country	Amikacin	Netilimycin	Fosfomycin	Gentamicin	Tobramycin	Doxycyline	Tetracycline	Minocycline	Tigecycline	Imipenem	Meropenem	Aztreonam	Mezlocillin	Carbenicillin	Piperacillin	Amoxicillin/ Clavulanate	Piperacillin/ Tazobactam	Ticarcillin	Ticarcillin/ Clavulanate	Trimethoprim/ Sulfamethoxazole	Cefoperazone/ Sulbactam	Nitrofurantoin	Cefalothin	Cefoxitin	Cefazolin	Ceftriaxone	Ceftazidime	Ceftazidime/ Clavulanate	Cefuroxime	Cefepime	Cefoperazone	Cefotaxime	Ampicillin	Ampicillin/ Sulbactam	Gatifloxacin	Levofloxacin	Ciprofloxacin	Moxifloxacin	Chloramphenicol	Flomoxef	Rifampicin	Colistin
Case 1 (Pasadakis et al. [9])	Greece	-	-	-	-	S	-	-	-	-	-	-	-	-	-	-	-	-	-	-	-	-	-	-	-	-	-	S	-	-	-	-	-	-	-	-	-	-	-	-	-	-	-
Case 2 (Seve et al. [10])	France	R	-	-	-	-	-	-	-	-	S	-	-	-	-	R	-	-	-	-	-	-	-	-	-	-	-	R	-	-	R	-	-	-	-	-	-	S	-	-	-	-	-
Case 3 (Chi et al. [11])	Taiwan	S	-	-	S	S	S	-	-	-	S	-	S	-	-	-	-	S	-	-	S	-	-	-	-	R	R	R	-	S	S	R	S	R	-	-	-	S	-	S	-	-	-
Case 4 (Han et al. [3])	USA																																										
Case 4a	USA	S	-	-	-	-	-	-	-	-	S	-	-	-	-	-	-	-	-	S	S	-	-	-	-	-	S	R	-	-	R	-	-	I	-	R	R	R	-	-	-	-	-
Case 4b	USA	S	-	-	-	-	-	-	-	-	S	-	-	-	-	-	-	-	-	S	S	-	-	-	-	-	R	R	-	-	R	-	-	R	-	R	R	R	-	-	-	-	-
Case 4c	USA	S	-	-	-	-	-	-	-	-	S	-	-	-	-	-	-	-	-	S	R	-	-	-	-	-	-	R	-	-	-	-	-	R	-	-	-	R	-	-	-	-	-
Case 4d	USA	S	-	-	-	-	-	-	-	-	S	-	-	-	-	-	-	-	-	S	R	-	-	-	-	-	-	S	-	-	-	-	-	I	-	-	-	R	-	-	-	-	-
Case 4e	USA	S	-	-	-	-	-	-	-	-	S	-	-	-	-	-	-	-	-	S	R	-	-	-	-	-	I	R	-	-	R	-	-	R	-	R	R	R	-	-	-	-	-
Case 4f	USA	S	-	-	-	-	-	-	-	-	S	-	-	-	-	-	-	-	-	S	R	-	-	-	-	-	R	R	-	-	R	-	-	I	-	R	R	R	-	-	-	-	-
Case 4g	USA	S	-	-	-	-	-	-	-	-	S	-	-	-	-	-	-	-	-	S	R	-	-	-	-	-	R	R	-	-	R	-	-	R	-	R	R	R	-	-	-	-	-
Case 5 (Lee et al. [12])	Taiwan																																										
Case 5a	Taiwan	S	-	-	-	-	-	-	-	-	-	-	-	-	-	-	-	S	-	-	-	-	-	-	-	-	-	-	-	-	-	-	-	-	-	-	-	R	-	-	-	-	R
Case 5b	Taiwan	S	-	-	-	-	-	-	-	-	-	-	-	-	-	-	-	S	-	-	-	-	-	-	-	-	-	-	-	-	-	-	-	-	-	-	-	R	-	-	-	-	R
Case 5c	Taiwan	S	-	-	-	-	-	-	-	-	-	-	-	-	-	-	-	S	-	-	-	-	-	-	-	-	-	-	-	-	-	-	-	-	-	-	-	R	-	-	-	-	R
Case 6 (Almuzara et al. [13])	Argentina	R	-	-	R	-	-	-	S	S	R	R	R	-	-	-	-	R	-	-	R	-	-	R	R	-	-	R	-	-	R	-	R	R	R	-	-	R	-	-	-	-	R
Case 7 (Pandit et al. [4])	USA	S	-	-	S	S	-	-	-	-	-	-	-	-	-	-	-	-	-	-	-	-	-	-	-	-	-	R	-	-	-	-	R	R	-	-	-	R	R	-	-	-	-
Case 8 (Lu et al. [14])	China	S	-	-	S	-	-	S	-	-	S	S	R	-	-	-	-	S	-	-	R	S	-	-	-	-	-	R	-	-	R	-	-	-	-	-	R	R	-	S	-	-	-
Case 9 (Shobha et al. [15])	India	S	-	-	-	-	-	-	-	-	S	-	-	-	-	-	S	-	-	S	S	-	-	-	-	-	-	-	-	-	S	-	S	-	-	-	-	R	-	-	-	-	-
Case 10 (Mahapatra et al. [16])	India	S	-	-	-	-	-	-	-	-	S	-	-	-	-	-	R	S	-	-	-	S	-	-	-	-	-	-	-	-	-	-	S	-	-	-	R	R	-	-	-	-	-
Case 11 (Cao et al. [17])	China	S	-	-	S	R	-	-	-	-	S	-	R	-	-	-	-	S	-	-	S	-	-	-	-	S	S	S	-	-	S	-	-	S	S	-	S	S	-	-	-	-	-
Case 12 (Ghosh et al. [18])	India	-	-	-	-	-	-	-	-	-	-	-	-	-	-	-	-	-	-	-	-	-	-	-	-	-	-	-	-	-	-	-	-	-	-	-	-	-	-	-	-	-	-
Case 13 (Chandra et al. [19])	India	S	-	-	S	-	-	-	S	S	S	S	-	-	-	-	-	-	-	-	S	S	-	-	-	-	-	S	-	-	S	S	-	-	-	S	S	S	S	-	-	-	R
Case 16 (Lupande-Mwenebitu et al. [20])	Democratic Republic of Congo	S	-	S	R	-	-	-	-	-	S	S	-	-	-	-	-	S	R	R	R	-	R	-	-	-	-	R	-	-	S	-	-	-	-	-	-	R	-	-	-	S	-
Case 17 (Burch et al. [5])	USA	-	-	-	-	-	-	-	-	-	-	S	-	-	-	-	-	-	-	-	-	-	-	-	-	-	R	-	-	-	-	-	-	-	-	-	-	-	-	-	-	-	-
Case 18 (Hassan et al. [6])	USA	-	-	-	-	-	-	-	-	-	-	-	-	-	-	-	-	-	-	-	-	-	-	-	-	-	-	-	-	-	-	-	-	-	S	-	-	-	-	-	-	-	-
Case 19 (Ferreira Caceres et al. [7])	USA	S	-	-	S	S	-	S	-	-	-	-	S	-	-	-	-	S	-	-	S	-	-	-	-	-	S	-	-	-	S	-	-	-	-	-	S	S	-	-	-	-	-
Case 20 (Thareja et al. [8])	USA	S	-	-	S	S	-	-	-	-	-	-	-	-	-	-	-	S	-	-	-	-	-	-	-	-	S	R	-	-	I	-	-	-	-	-	-	R	-	-	-	-	-
Case 21 (current)	USA	S	-	-	S	S	-	-	-	-	-	S	-	-	-	-	-	S	-	-	S	-	-	-	-	-	-	-	I	-	S	-	-	-	-	-	I	R	-	-	-	-	-

## Conclusions

*B. diminuta* is a rare cause of clinically significant infection, typically associated with malnourished or immunocompromised states. Antimicrobial resistance is a significant challenge to practitioners treating *B. diminuta* infections. Analysis reveals extensive resistance to fluoroquinolones, sulfonamides, and colistin. Sensitivity is preserved to carbapenems, aminoglycosides, and ticarcillin/clavulanate. Varied sensitivity to beta-lactams is observed, highlighting the need for antibiotic sensitivity assays when a clinically significant infection is encountered.

Here, we add to the existing literature a case of *B. diminuta* bacteremia with septic shock in an immunocompetent patient successfully treated with cefepime and build on previously reported cases with a compilation of antimicrobial resistance patterns emerged from a detailed analysis of available literature. Further research is warranted to explore the immunologic mechanisms behind *B. diminuta* infections in immunocompetent patients.
